# A Comprehensive Study of Recent Path-Planning Techniques in Dynamic Environments for Autonomous Robots

**DOI:** 10.3390/s24248089

**Published:** 2024-12-18

**Authors:** Nour AbuJabal, Mohammed Baziyad, Raouf Fareh, Brahim Brahmi, Tamer Rabie, Maamar Bettayeb

**Affiliations:** 1 Research Institute of Sciences & Engineering, University of Sharjah, Sharjah P.O. Box 27272, United Arab Emirates; nabujabal@sharjah.ac.ae (N.A.); mbaziyad@sharjah.ac.ae (M.B.); 2 Electrical Engineering Department, University of Sharjah, Sharjah P.O. Box 27272, United Arab Emirates; mbettayeb@sharjah.ac.ae; 3 Department of Electrical and Electronics Engineering, Collège Ahuntsic, Montreal, QC H2M 1Y8, Canada; 4 Computer Engineering Department, University of Sharjah, Sharjah P.O. Box 27272, United Arab Emirates; trabie@sharjah.ac.ae

**Keywords:** path planning, multi-robot, formation control, leader–follower, virtual formation, behavior-based formation, dynamic formation, centralized decision, decentralized decision, distributed decision, hybrid decision

## Abstract

This paper presents a comprehensive review of path planning in dynamic environments. This review covers the entire process, starting from obstacle detection techniques, through path-planning strategies, and also extending to formation control and communication styles. The review discusses the key trends, challenges, and gaps in current methods to emphasize the need for more efficient and robust algorithms that can handle complex and unpredictable dynamic environments. Moreover, it discusses the importance of collaborative decision making and communication between robots to optimize path planning in dynamic scenarios. This work serves as a valuable resource for advancing research and practical applications in dynamic obstacle navigation.

## 1. Introduction

Path planning is a critical process in multi-robot systems, focusing on finding an optimal, obstacle-free path to a destination while considering the robot’s capabilities and environmental constraints [1,2]. Key factors in evaluating path-planning algorithms include path length, computational speed, smoothness, energy cost, and safety. Optimizing these factors simultaneously is challenging, as higher map resolutions improve path quality but also increase processing time.

The previously described trade-off problem between execution speed, path quality, and safety is expected to be more challenging in an environment with dynamic obstacles, such as pedestrians, vehicles, or animals. The robot must adapt its path in real time to avoid collisions with moving obstacles while minimizing deviations from the optimal route. Since the behavior of these moving objects is unpredictable, algorithms need to consider their velocity and trajectory, predicting future positions for safe navigation. This real-time adjustment increases the computational cost, requiring more sophisticated algorithms to balance speed, path quality, and safety.

The efficient detection of moving obstacles is an important preliminary step for path-planning techniques. Sensors such as LiDAR, ultrasonic, stereo cameras, infrared, and radar are typically used to measure the distance and location of obstacles. Once detected, path-planning algorithms compute a collision-free path, either by maneuvering around or through the obstacles. To address these challenges, numerous algorithms have been proposed, aiming to find the optimal trade-off between various factors. These algorithms utilize different optimization techniques, such as heuristic search, sampling-based methods, and reinforcement learning, to generate the optimal paths.

There have been several attempts to produce reviews of path-planning techniques for autonomous robot systems in a dynamic environment. However, the reviews published recently in the literature were limited to either reviewing only a specific robot type, such as Unmanned Ground Vehicle (UGV) systems [3] and Autonomous Underwater Vehicle (AUV) systems [4], or neglecting the obstacle detection methods, which are essential when dealing with dynamic obstacles, and focusing on the challenges of path planning in dynamic environments [5,6]. Some reviews focused on either detecting dynamic obstacles [7] or avoiding them [8]. This paper presents a comprehensive review that combines the key topics related to dynamic obstacles in autonomous robot systems, including obstacle detection, path planning, and formation control. To the best of the authors’ knowledge, there is no recent comprehensive study on state-of-the-art multi-robot systems in dynamic environments. This review aims to provide a unified perspective on how these critical aspects interact in dynamic environments. Specifically, dynamic obstacles are classified into types, such as randomly moving, cooperative, and unconscious obstacles. Additionally, this paper explores the communication and collaboration requirements of multi-robot systems during navigation. It classifies autonomous robot systems based on their “communication style” into centralized, decentralized, and distributed approaches, offering insights into how these systems manage coordination and decision making in complex environments.

The rest of this paper is organized as follows. Section 2 provides a summary of the methods used in the literature to detect obstacles. A classification based on the path-planning techniques used is presented in Section 3. Another classification, based on the obstacle consciousness, is presented in Section 4. Section 5 classifies the techniques based on communication style, while Section 6 classifies the techniques based on obstacle shape. Section 7 shows some empirical experiments to support the findings discussed in this paper. A discussion on the gaps and challenges in path planning for dynamic obstacles is presented in Section 8. Finally, the conclusions are presented in Section 9.

## 2. Obstacle Detection Methods

In this paper, obstacle detection methods are categorized into range-based and vision-based approaches. Range-based methods, using sensors such as LiDAR, radar, and ultrasonic, offer accurate measurements but can be affected by environmental noise. Vision-based methods analyze camera images but are sensitive to lighting and occlusions. Comparative analyses of sensing technologies and recent techniques are provided in Table 1 and Table 2. Section 2.1 covers range-based methods for dynamic obstacles, while Section 2.2 focuses on vision-based methods.

### 2.1. Range-Based Methods

**Figure 1:**
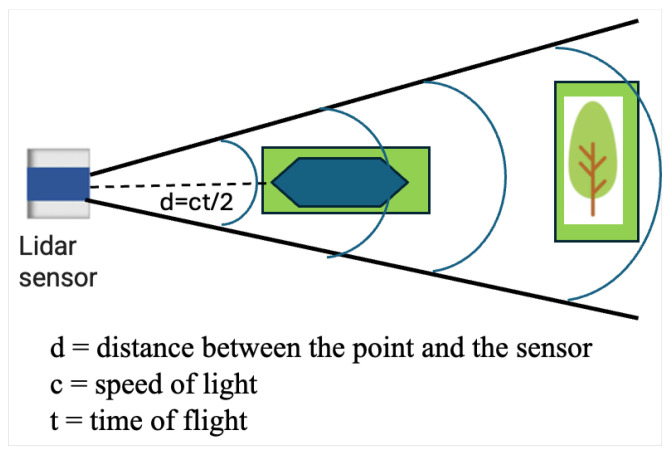
Range -based methods.

Range-based methods typically use technologies such as ultrasonic or radar sensors to send out signals that bounce off objects and return to the sensor. By analyzing the time it takes for the signal to return, range-based methods can calculate the distance to the object and provide information about its size and location as mentioned in Figure 1. These methods are fast, accurate, and useful for detecting obstacles in real time. Moreover, they can be effective in a range of settings, including industrial automation, transportation, and defense applications. However, they are limited in their ability to detect small or low-lying objects that are not within their range, and they are affected by environmental factors such as dust, smoke, and fog.

**Table 2:** Recent works focusing on different obstacle detection methods.

Class	Sensor	Paper	Year	Range of Detection and Degree	Detection Speed (Obstacle Speed)	2D\3D	Comment
Range-Based Methods	LiDAR	[9,10,11]	2018, 2020, 2018	[9] 10–50 m. [10] Up to 20 m. [11] At least 1 m away.	[9] Below 20 km/h. [10] Sim: 0.4–1.2 m/s; Exp: 0.5 m/s. [11] 1 m/s.	[9,11] 3D. [10] 2D.	[11] Experimentally tested on a differential two-wheel drive system to track a person.
	Ultrasonic	[12,13]	2019, 2017	[12] 0–100 cm. [13] 0.02–7.00 m.	[12] – [13] Static obstacles.	[12] 2D. [13] –	Used to aid visually impaired persons.
	Radar	[14,15,16]	2021, 2021, 2015	[14] Up to 175 m. [17] Limited range. [16] Moderate to high range.	[14] Limited speed. [17] Moderate. [16] Relatively fast.	2D	[14] Designed for USVs. [16] The detection speed may be limited because of clustering and the COBWEB algorithm.
	Infrared	[18]	2021	Maximum distance of 5 m.	Very inaccurate	2D	Used to aid visually impaired persons; average deviation of 1.23 m.
	Ladar	[16]	2015	Obstacle 1 > 15.62 m, obstacle 2 > 23.63 m.	Not specified	2D	Driverless cars.
	Multi-sensor	[19,20,21,22,23]	2021, 2017, 2017, 2017, 2016	[19] 3 m in 3 different directions. [20] 120 m and 360 degrees. [21] 180 degrees. [22] ∼16 cm. [23] 4–40 cm infrared, 15–650 cm (70) ultrasonic.	[19,21,22,23] Static obstacles. [20] Not specified.	[19,21] 2D. [20] 3D. [22,23] –.	[19] Ultrasonic + infrared + water sensor for visually challenged people. [20] + Wireless sensor. [21,22,23] Ultrasonic + infrared.
Vision-based methods	Stereo	[24,25]	2020, 2020	[24] 1.5–2 m. [25] 5 m.	[24] 100 mm/s and 20 degree/s. [25] Above 0.45 m/s is dynamic and less than that is static.	[24] 3D. [25] 2D and 3D.	[24] Integrated a fuzzy logic design. [24,25] Designed for mobile robots.
	RGB-D	[17,26]	2022, 2018	[26] Max distance of 3.669 m. [17] –	[26] Maximum speed of 5 m/s; cannot detect very fast obstacles. [17] –	3D	[26] Designed for mobile robots. [17] Designed for blind people.
	YOLOv3	[27]	2020	–	Real time	2D	Used YOLOv3 to detect dynamic obstacles in real time, combined with DWA approach.
Hybrid methods	Vision and Range	[28,29,30,31]	2021, 2019, 2019, 2018	[28] Radius of 12 m and scanning range of 360 degrees. [29] – [30] – [31] 0–174 m radar, 0–60 m, 1–100 m thermal camera, 1.5–5 m stereo camera.	[28] Low moving speed. [29] Normal speed on the highway. [30] Wind speeds of up to 5.8 m/s. [31] Approximately 70,000 points/frame.	[28,30,31] 3D. [29] 2D.	[28] + Monocular camera. [29] + Camera. [30] Stereo camera + optical flow sensor + sonar sensor for UAVs. [31] + Radar + stereo + thermal cameras for mobile robots.

LiDAR (Light Detection and Ranging) sensors use laser beams to detect obstacles in their surroundings. These sensors emit light pulses that bounce off objects and return to the sensor, allowing it to create a 3D map of the surrounding environment. LiDAR sensors can detect the distance, size, and shape of objects while navigating, allowing for online detection and efficient collision avoidance. LiDAR sensors have proven to be highly effective in detecting obstacles in a wide range of environments, including in low-light conditions or in the presence of fog or rain. As a result, LiDAR sensors have become an essential component of many advanced technologies that rely on accurate obstacle detection. In [9], a dynamic obstacle detection system with a detection range of 10–50 m was introduced, suitable for detecting obstacles when a vehicle is moving at speeds below 20 km/h. Similarly, a real-time dynamic obstacle detection system with a range of up to 20 m was developed utilizing LiDAR by the authors of [10]. In addition, an obstacle detection method using LiDAR was presented in [11], effective at a minimum distance of 1 m.

Refs. [9,10,11], while presenting valuable advancements in dynamic obstacle detection and tracking for autonomous robots, each has notable shortcomings. The work in [9] relied heavily on nearest-neighbor clustering and the Multi-Hypothesis Tracking (MHT) algorithm, which may struggle with fast-moving obstacles or real-time performance in complex, dynamic environments. Onda et al.’s work [11] on 3D LiDAR with a wide vertical field of view lacked detailed shape recognition, limiting its ability to accurately detect obstacles in close proximity, which is crucial for precise navigation in urban areas. Dong et al.’s approach [10], using 2D LiDAR and a Kalman filter for dynamic avoidance, achieved good results in indoor environments, but the approach may struggle in more cluttered or larger-scale scenarios due to its reliance on simplified geometric models and the potential computational cost of merging and classifying point clouds. All three methods need further refinement to address scalability, real-time processing, and handling highly dynamic environments effectively.

On the other hand, ultrasonic sensors emit high-frequency sound waves that bounce off nearby objects and return to the sensor. The time it takes for the sound waves to return to the sensor is used to calculate the distance to the object. Ultrasonic sensors can detect obstacles within a range of several meters and are commonly used in robotics, industrial automation, and automotive applications. They are particularly useful for detecting large or flat objects, as well as for detecting objects in environments with low visibility. However, ultrasonic sensors may struggle in environments with a lot of acoustic interference or when detecting small or irregularly shaped objects. Despite these limitations, ultrasonic sensors remain a popular choice for obstacle detection due to their low cost, ease of use, and reliability.

The authors of [12] developed an intelligent system using ultrasonic sensors with a detection range of 0–100 cm, suitable for close-range obstacle detection. Another system was presented in [13] with a range of 0.02 m to 7.00 m, primarily for static obstacle detection. These techniques are useful for applications that require proximity detection, such as assisting visually impaired individuals. However, both systems present significant shortcomings. The system in [13], which relies on ultrasonic sensors, struggles with accuracy due to interference from motion artifacts. Similarly, the system in [12], despite integrating ultrasonic sensors, voice navigation, and GPS, has a narrow detection range (up to 100 cm) and fails to adapt in real time to environmental changes. This makes both systems impractical for many real-world applications where obstacles and conditions are constantly changing.

Radar (Radio Detection and Ranging) sensors use radio waves to detect obstacles in their environment [14]. Radar sensors can detect obstacles within a range of several hundred meters and are particularly effective at detecting moving objects. This technology is commonly used in automotive applications, air traffic control, and military operations. Radar sensors can also operate in a wide range of environmental conditions, including low-visibility situations or during inclement weather. Despite their effectiveness, radar sensors may struggle to detect small or stationary objects, which makes them less suitable for certain applications. However, radar sensors remain a popular choice for obstacle detection due to their reliability and versatility.

While the approach presented in [15] is robust in environments with dust and other weather-related challenges, it suffers from a limited radar range and significant blind spots, which hinder its ability to detect obstacles around the vehicle. Additionally, the reliance on sparse data for dynamic obstacle detection could result in missed detections or delayed responses in real-time scenarios. On the other hand, the authors of [16] focused on laser radar and the use of advanced algorithms such as COBWEB for road edge detection. However, the approach has computational inefficiencies due to complex clustering and a lack of robustness in irregular terrain conditions. It also relies heavily on specific hardware, which may not be universally applicable. Lastly, the authors of [14] presented a promising system using radar and ENC, but it struggles with high-speed object detection and short-range obstacles, making it less effective for close-proximity navigation. Moreover, the radar data’s limited update rate could cause delays in dynamic obstacle tracking.

Infrared sensors use infrared light to detect objects and their proximity. The authors of [18] presented an optimized infrared-based obstacle detection system with a maximum detection distance of 5 m. However, infrared sensors can be relatively inaccurate, and their effectiveness can be influenced by environmental conditions. These systems are frequently employed to assist visually impaired individuals and navigate in low-light conditions. The authors of [18] presented a system that has several limitations. The RSS-based localization method has an average deviation of 1.23 m, making it inaccurate for real-world applications. The infrared sensor used for obstacle detection has a limited range (5 m) and cannot reliably estimate distances or detect multiple objects effectively. While it ensures anonymity, the system lacks the precision needed for dynamic, fast-changing environments, making it unsuitable for real-time navigation, especially for visually impaired users.

Multi-sensor systems integrate various sensor types to enhance obstacle detection capabilities. Several studies have explored the use of multi-sensor setups, combining sensors such as ultrasonic and infrared sensors. One study presented an intelligent system that utilized ultrasonic, infrared, and water sensors to aid visually challenged individuals [19]. Another work employed a combination of LiDAR and wireless sensors for its obstacle detection system [20]. An additional research effort utilized a multi-sensor setup that included ultrasonic and infrared sensors to detect obstacles within a range of 3 m. Similarly, a system has been developed that incorporates ultrasonic and infrared sensors, providing obstacle-detection capabilities for mobile robots, with a proximity range of approximately 16 cm. Lastly, another study used a combination of ultrasonic and infrared sensors for obstacle detection within specific ranges. These multi-sensor systems offer the advantage of fusing data from multiple sources to improve obstacle detection accuracy and reliability, making them suitable for a variety of applications.

### 2.2. Vision-Based Methods

Vision-based methods rely on the capture of visual data, which are analyzed by computer algorithms to identify objects based on their color, shape, and texture. Vision-based methods can provide more detailed information about the surrounding environment compared to range-based methods, as they can detect objects of various sizes and shapes, including those that are not within the range of sensors. They can also be used to detect and classify different types of obstacles, such as pedestrians, vehicles, and traffic signs. However, they may not perform well in low-light conditions, and their performance can be affected by factors such as camera calibration and image quality. In addition, these methods require a high degree of accuracy to ensure safe navigation in environments with complex or moving obstacles.

Stereo cameras use two or more lenses to capture depth information about objects in their field of view. By analyzing the differences in perspective between the images captured by each lens, stereo cameras can create a 3D map of their environment. Stereo cameras are particularly effective at detecting small and irregularly shaped objects, as well as objects that are far away. However, stereo cameras can struggle in low-light conditions or when there are obstructions blocking the camera’s view. Despite these limitations, stereo cameras are widely used in advanced driver assistance systems (ADAS), autonomous vehicles, and drones, where accurate obstacle detection is essential.

In recent studies on stereo vision technology, researchers have utilized this method for obstacle detection with a range of 1.5 to 2 m, demonstrating its ability to handle dynamic obstacles with speed variations of up to 100 mm per second and 20 degrees per second [24,25]. The approach also incorporates 3D modeling, enhancing its suitability for mobile robot applications [24,25]. However, both systems face challenges. The method in [24], while using a low-cost stereo vision setup and fuzzy logic for obstacle avoidance, struggles with high computational demands, limiting its real-time performance on resource-constrained platforms. Similarly, the approach in [25], which integrates stereo camera data and a people detector for dynamic object tracking, suffers from a reliance on noisy stereo data and computational overhead, impacting its efficiency on platforms with limited processing power.

An innovative approach to obstacle detection has been introduced using RGB-D cameras [17,26]. The system efficiently identifies obstacles within a maximum distance of 3.669 m [26] and can operate at speeds of up to 5 m per second [26]. However, it has limitations in detecting very fast-moving obstacles [26]. The 3D modeling capabilities of the system make it a valuable choice for various applications, especially those involving mobile robots [17,26].

A novel approach for obstacle detection was implemented using the YOLOv3 (You Only Look Once version 3) algorithm, as detailed in [27]. This method allows for the real-time detection of dynamic obstacles, making it highly effective in environments where obstacles are constantly changing or moving. YOLOv3’s capability to identify objects with high accuracy in a single pass through the network enables fast processing times, which are crucial for applications requiring immediate responses. To further enhance the obstacle-avoidance mechanism, YOLOv3 has been integrated with the Dynamic Window Approach (DWA), a well-known method for real-time robot motion planning [27]. The DWA optimizes the robot’s velocity within a dynamic window, taking into account both its current state and the detected obstacles. The combined system uses a 2D detection approach, which allows for quick identification of obstacles in the robot’s path, providing a rapid response to potential collisions. This integration is particularly beneficial in fast-paced and dynamic environments, such as warehouses or autonomous vehicles navigating busy streets, where real-time decision making is essential to ensure safety and efficiency [27].

**Figure 2:**
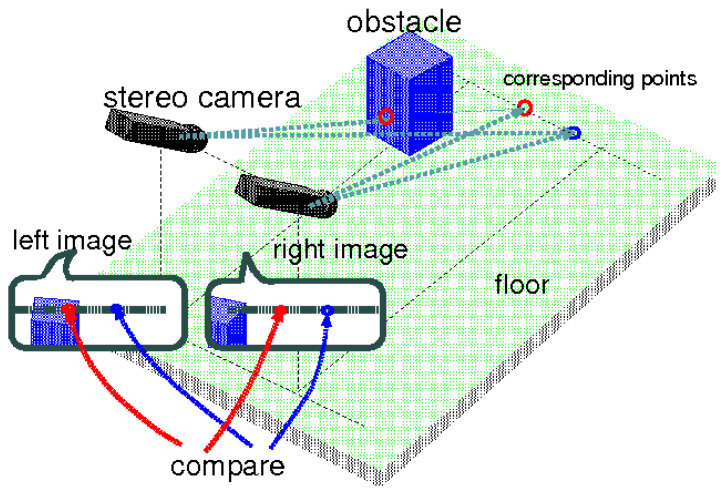
Stereo cameras.

Hybrid obstacle detection methods have demonstrated their potential in various scenarios. In one approach, a high-level fusion technique combined LiDAR and a monocular camera for low-speed obstacle detection and avoidance [28]. Another method integrated LiDAR and a camera system to handle dynamic obstacles on highways [29]. For UAV applications, a fusion of stereo cameras, optical flow sensors, and sonar sensors proved effective in challenging environments [30]. In mobile robot applications, a multi-sensor fusion approach combined LiDAR, radar, and stereo and thermal cameras (Figure 2) [31].

## 3. Classification Based on Path-Planning Techniques

**Figure 3:**
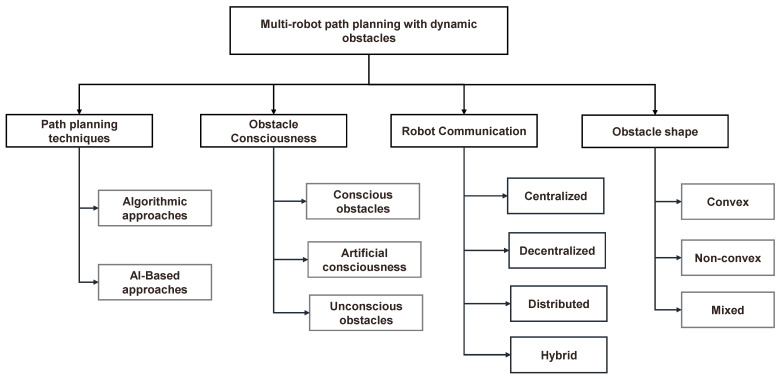
General classification of path-planning techniques for dynamic obstacles.

**Figure 4:**
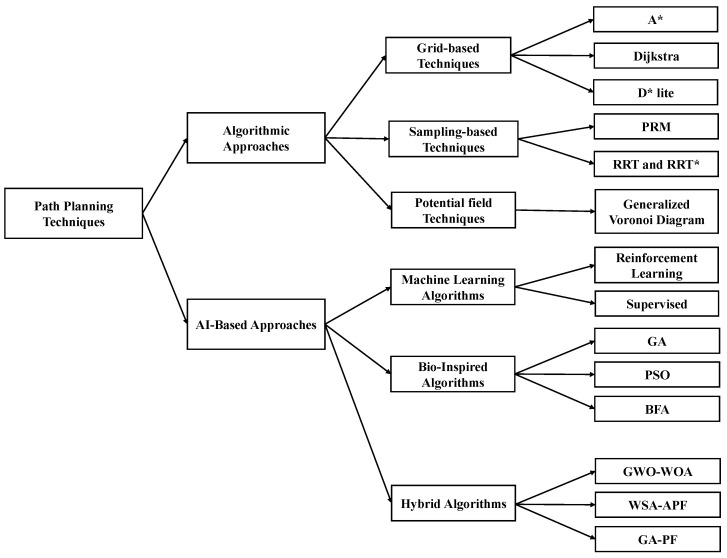
General classification of path-planning techniques.

This paper classifies recent works in path planning for moving obstacles based on various aspects, including the path-planning techniques used, obstacle consciousness, and obstacle shape. Figure 3 shows the different classifications used in this paper. This section elaborates on classification based on the technique used. Table 3 shows a summary of recent works based on the adopted algorithmic path-planning technique, while Table 4 shows recent works related to AI-based path-planning approaches. Figure 4 illustrates the classification of path-planning techniques.

**Table 3:** Classification based on algorithmic path-planning techniques.

Class	Technique	Complexity		Papers	Year	Validation		Comment
		Time	Space			Sim.	Exp.	
**Grid-based**	A*	O($b^{d}$)	O($b^{d}$)	[32,33,34,35,36]	2020, 2021, 2013, 2019, 2023	[33,35,36]	[32,34]	[34] Experiments on a small-sized version of a 5DPO robot. [35] Applied two manipulators, each one 6 DoF.
	Dijkstra	O($b^{d}$)	O($b^{d}$)	[37,38,39]	2018, 2013, 2020	[37,38]	[39]	[39] Tested experimentally on autonomous transportation systems (trains). [37] Applied to an AUV system. [38] Application for warehouse operations.
	D* Lite	O($b^{d}$)	O($b^{d}$)	[40,41,42,43,44,45]	2017, 2023, 2022, 2021, 2021, 2017	[40,41,43,45]	[42,44]	[40] Applied to a multi-agent autonomous swarm. [41] Application for a large disaster area. [42,43] Applied to a USV system. [44] A real-time path-planning algorithm implemented on a UAV system.
**Sampling-based**	PRM	O($n^{2}$)	O(n)	[46,47,48,49]	2014, 2021, 2013, 2022	[46,47,49]	[48]	[46,49] Used PRM with Q-learning. [47] Application for autonomous UAV exploration. [48] Temporal probabilistic roadmap implemented on two UAV systems.
	RRT	O(k)	O(k)	[50,51,52]	2022, 2015, 2015	[50,51,52]		[50,52] Applied to a multi- UAV system.
	RRT*	O(k)	O(k)	[53,54,55]	2019, 2020, 2022	[53,54,55]		[54] Applied to a UAV system. [55] Applied to a nonholonomic mobile robot.
**APF**	Generalized Voronoi Diagram	O(n log n)	O(n)	[56,57,58]	2022, 2021, 2021	[56,57,58]		[56,57] Applied to multiple UGV systems, taking path-priority order into account. [58] Application for smart warehouses.
	Others	-	-	[59,60,61]	2019, 2018, 2020	[59,60,61]		[59] Neighborhood APF approach. [60] Unknown Input Observer and Angle-Dependent APF algorithm. [61] Utilized a centralized architecture for multiple UGV systems.

### 3.1. Algorithmic Approaches

**Figure 5:**
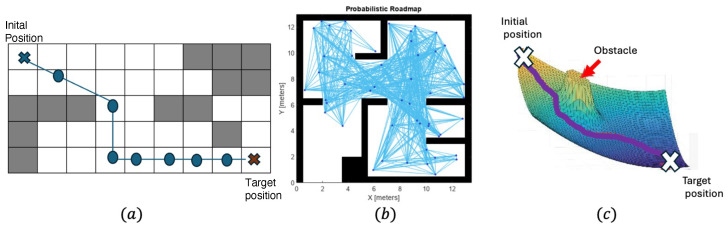
(**a**) Grid -based. (**b**) Sampling-based. (**c**) Potential field.

Algorithmic path-planning approaches were widely used for robot navigation before the emergence of artificial intelligence methods. These traditional techniques involve planning the movement of robots between two points using mathematical models and algorithms. Although algorithmic path-planning methods provide globally optimal paths and are easy to implement, they can struggle to handle dynamic and unpredictable environments and may be computationally expensive. Furthermore, they may not be able to quickly respond to unexpected obstacles or adapt to changes in the environment [62]. Nevertheless, algorithmic path-planning approaches remain relevant and provide a valuable foundation for modern path-planning techniques. In this paper, as shown in Figure 5, algorithmic path-planning approaches are classified into three main subcategories: grid-based techniques, sampling-based techniques, and artificial potential field (APF) techniques.

Algorithms can be evaluated based on their complexity level. Table 3 displays the time and space complexity of each technique. The computational complexities of path-planning algorithms differ significantly, reflecting their respective strengths and weaknesses in various applications, especially in resource-constrained and real-time environments. A* and other grid-based techniques have an exponential time complexity of $O(b^{d})$ and a space complexity of $O(b^{d})$, where *b* is the branching factor and *d* is the depth of the search. This makes A* suitable for grid-based environments, but its high computational demands render it less efficient for large or high-dimensional spaces, particularly when real-time performance is required. PRM, a sampling-based algorithm, with a time complexity of $O(n^{2})$ and a space complexity of O(n), performs well in high-dimensional spaces but can struggle in cluttered environments due to the need for extensive sampling. RRT, with linear time and space complexities of O(k), offers faster planning and is better suited for real-time applications, as it incrementally explores the space. However, this speed comes at the cost of optimality, often requiring refinement through methods such as RRT* to generate smoother paths.

**Table 4:** Classification based on adopted AI-based path-planning approaches.

Class	Technique	Papers	Year	Validation		Comment
				Sim.	Exp.	
**Bio-inspired**	GA	[63,64]	2020, 2021	[63,64]		[63] Multiple UGV systems. [64] Applied in a 3D live environment for viscoelastic bio-particles.
	PSO	[65,66,67,68,69]	2021, 2018, 2017, 2021, 2021	[65,66,67,68,69]		[65,66,67] Applied to multiple UGV systems. [68] Applied to a 10-DOF rocker-bogie-type wheeled mobile robot (Rover). [69] 3D path planning for multi-UAVs
	Bacterial Foraging	[70,71,72]	2013, 2018, 2015	[70,71,72]		[70,71] Applied to multiple UGV systems.
**Machine Learning**	Supervised	[73,74,75]	2022, 2021, 2024		[73,74,75]	[73] A deep learning approach based on the CNN model. [74] Trajectory prediction model based on RNNs. [75] Designed for narrow streets; used Generative Adversarial Networks (GANs).
	RL	[76,77,78,79,80,81,82]	2020, 2020, 2022, 2020, 2022, 2023, 2024	[76,78,80,81,82]	[77,79]	[77] Proposed a globally guided reinforcement learning approach. [78] Used the state-coded deep Q-network (SC-DQN) algorithm for unmanned helicopters. [79,80] Designed for multi-UGV systems. [81] Optimized the parameter update and exploration strategies of the actor-critic algorithm. [82] Traffic-type independent training environment
**Hybrid Techniques**	A* and CSA-APF	[83]	2024	[83]		[83] Incorporated techniques such as a weighted heuristic function, cubic B-spline curves, and a CSA-APF algorithm with simulated annealing. [84] An extra evaluation function was introduced to mitigate the problem of local optima in complex environments. [85] A cooperative hunting multi-robot system was studied when the target moves with unknown behavior. [86] The sampling process of rapidly exploring random trees was guided using the potential field to accelerate the convergence rate of the rapidly exploring random tree to low-cost obstacle-avoidance paths. [87] Distributed path planning and obstacle avoidance in unknown complex terrains.
	A*-DWA	[84]	2024	[84]		
	GWO-WOA	[88]	2022	[88]		
	WSA-APF	[85]	2022	[85]		
	APF-BEA	[89]	2015	[89]		
	Max–Min ACO-D*	[90]	2018	[90]		
	RRT-APF	[86]	2022	[86]		
	ACO-DWA	[87]	2022	[87]		
	NPQ-RRT*	[91]	2021	[91]		
	ABC-EP	[92]	2018	[92]		
	A*-Dijkstra	[93]	2014	[93]		
	A*-Dijkstra	[94]	2017	[94]		
	GA-PF	[95]	2020	[95]		

The relation to real-time performance becomes critical in dynamic and high-dimensional scenarios, where the ability to adapt quickly to changes is essential. A*’s computational overhead makes it less viable for dynamic, time-sensitive tasks, whereas PRM and RRT, due to their lower complexities, are more appropriate for real-time applications. RRT, in particular, strikes a balance between speed and adaptability, making it a popular choice for real-time dynamic path planning, although its suboptimal paths may require post-processing. Ultimately, the selection of an algorithm for real-time performance depends on the specific environment and the trade-off between computational efficiency, optimality, and adaptability to dynamic changes.

#### 3.1.1. Grid-Based Techniques

Grid-based path planning is an algorithmic approach that divides the environment into a grid of cells, assigning movement costs to each cell to compute the optimal paths using algorithms such as A*, Dijkstra, and D* Lite. A* improves search efficiency with heuristics, while D* Lite adapts to environmental changes, making this method effective in dynamic environments [40]. However, grid-based techniques face challenges in handling large environments due to high computational costs.

Grid-based methods have been influential in the area of obstacle avoidance in robotics [32,33,34,35,36]. Researchers have employed these methods in various studies, with validation methods encompassing both simulation and experimentation. Specific implementations include notable cases where small-sized versions of robots or systems with multiple manipulators were used. For instance, one study employed a small-sized version of a robot [34], while another applied grid-based methods to systems with multiple manipulators, each having 6 degrees of freedom [35]. In another study, these methods were applied in diverse contexts such as autonomous transportation systems, autonomous underwater vehicles [37], and warehouse operations [38]. In the case of another grid-based method, it was applied in various scenarios, including multi-agent autonomous swarms [40], large disaster areas [41], unmanned surface vehicle (USV) systems [42,43], and unmanned aerial vehicle (UAV) systems [44].

#### 3.1.2. Sampling-Based Techniques

Sampling-based path planning is a widely used approach that generates paths by randomly sampling the environment and constructing a graph connecting valid points. Algorithms such as probabilistic roadmap (PRM), rapidly exploring random tree (RRT), and RRT* are common, where PRM builds a roadmap of low-cost paths, RRT explores unexplored regions through tree expansion, and RRT* optimizes paths with a cost function [96]. These techniques are well suited for complex and high-dimensional environments, adaptable to dynamic obstacles, and computationally efficient. However, challenges include the potential for suboptimal paths and heavy reliance on effective sampling strategies.

The combination of PRM and Q-learning has been widely applied in robotics, particularly for UAV exploration, offering a robust framework for path planning in dynamic environments. PRM provides a reliable structure for global path planning, while Q-learning enhances this by adapting to environmental changes in real time, improving decision making based on prior experiences [46,47]. However, one potential shortcoming of this approach lies in its scalability when applied to larger, more complex environments. As the number of obstacles and agents increases, the computational load required for continuous learning and decision making can become prohibitive, limiting its applicability in real-time, large-scale applications.

The study in [48] introduced Temporal-PRM, an extension of the probabilistic roadmap (PRM) algorithm that incorporated the concept of time to enable dynamic obstacle avoidance. Temporal-PRM augmented the PRM structure with a time-aware graph and employed a modified A* search algorithm to efficiently query paths while maintaining PRM’s ability to handle multiple queries and produce smooth paths. This approach achieved collision avoidance in highly dynamic environments with minimal computational overhead. Experimental results demonstrated that Temporal-PRM outperformed state-of-the-art sampling-based planners in cluttered, dynamic scenarios and successfully performed real-time obstacle avoidance onboard flying robots.

On the other hand, RRT and RRT* techniques, as employed in UAVs and nonholonomic mobile robots, provide a highly flexible solution for dynamic obstacle avoidance, allowing robots to adjust their paths quickly in response to environmental changes [50,51,53]. The ability of RRT to navigate in real time and the optimality guarantees offered by RRT* are significant advantages, particularly in mission-critical tasks. However, these techniques suffer from inefficiency in high-dimensional spaces or when obstacles are densely packed, as RRT may take longer to find feasible paths in such scenarios. Additionally, RRT* requires high computational resources to optimize the path continuously, which can limit its real-time effectiveness, particularly on platforms with constrained computational power.

Both PRM with Q-learning and RRT-based approaches offer valuable tools for dynamic path planning, but each has its own limitations. While PRM and Q-learning work well in structured environments, they struggle with real-time adaptability in highly dynamic or unpredictable settings. Similarly, while RRT and RRT* are effective in navigating cluttered environments and ensuring optimality, their performance can degrade in large, high-dimensional spaces or when computational resources are limited.

#### 3.1.3. Artificial Potential Field Techniques

Artificial potential field (APF) path planning is an algorithmic method that models the environment as a virtual potential field, where obstacles exert repulsive forces and the goal exerts an attractive force, guiding the robot along the gradient of the field. This technique is computationally efficient, handles dynamic obstacles effectively, and ensures collision avoidance. However, challenges include the risk of becoming stuck in local minima, difficulty navigating narrow passages, and difficulties in handling multi-goal scenarios. Despite these limitations, APF remains a valuable approach in robotics and has been extended in various ways to address these issues.

Researchers have applied various techniques within the APF framework to address unique challenges and environments, enhancing its adaptability and utility. One notable approach within the APF framework is the use of Generalized Voronoi Diagrams. The authors of [56,57], for instance, applied this technique extensively in scenarios involving multiple UGVs, considering path-priority order and efficiently guiding robots around obstacles. Additionally, ref. [58] employed the technique in smart warehouses, optimizing the navigation of robots in confined spaces. Researchers have also explored other techniques under the APF category, including those based on the neighborhood approach and input observers. These methods, as exemplified in [59,60], enable collision avoidance and efficient path planning in various contexts. For example, ref. [60] applied the Unknown Input Observer and Angle-Dependent APF algorithm to navigate mobile robots, demonstrating its effectiveness in steering robots safely through complex environments. Furthermore, ref. [61] utilized a centralized architecture based on APF in multiple UGV systems, emphasizing its versatility and practicality in real-world applications.

### 3.2. AI-Based Approaches

AI-based approaches can be classified into three broad categories: machine learning algorithms, bio-inspired algorithms, and hybrid algorithms.

#### 3.2.1. Bio-Inspired Algorithms

Bio-inspired algorithms, also known as nature-inspired algorithms, draw inspiration from biological systems, processes, or behaviors to solve complex problems. These algorithms simulate natural phenomena and use principles such as evolution and swarm intelligence. They aim to solve optimization, search, or decision-making problems. Genetic algorithms (GAs) are based on the concept of natural selection and evolution. They use techniques such as mutation, crossover, and selection to evolve a population of solutions over successive generations, ultimately converging toward an optimal or near-optimal solution. Particle swarm optimization (PSO) is inspired by the behavior of bird flocks or fish schools, and the PSO algorithm simulates the collective movement and cooperation of particles in a multidimensional search space. These algorithms iteratively adjust the positions and velocities of particles to find the optimal solution.

In the literature, GA-based techniques have been widely employed, demonstrating their efficacy in enhancing path planning for various multi-robot systems, as witnessed in multiple Unmanned Ground Vehicle (UGV) applications [63]. Notably, these techniques showcase their adaptability and effectiveness in navigating complex environments. Moreover, the utilization of GAs extends to 3D environments, where they contribute to path planning for viscoelastic bio-particles, further underscoring their versatility and applicability [64]. On the other hand, the PSO technique has gained traction in path planning for robotics, with its implementation extending to various contexts [65,66,67,68,69]. Researchers have harnessed the power of PSO to address the path-planning challenges presented by multiple UGV systems. Additionally, PSO has been employed to optimize path planning for a 10-degree-of-freedom (DOF) rover-like mobile robot, showcasing its adaptability in diverse robotic scenarios [65]. Furthermore, PSO has played a pivotal role in 3D path planning for multiple unmanned aerial vehicles (UAVs), highlighting its utility in complex missions [66,67,68,69]. The Bacterial Foraging Algorithm (BFA) is yet another bio-inspired approach that has found its place in robotic path planning [70]. This technique has been effectively applied in scenarios involving multiple UGVs, contributing to their ability to navigate complex environments. Its application underscores the algorithm’s adaptability and utility in addressing challenges related to robotic path planning in dynamic and uncertain environments [71,72]. However, the techniques in [71,72] suffer from limitations such as sensitivity to parameter settings and the potential for slower convergence in large-scale environments. These challenges can be typically addressed by fine-tuning the algorithm’s parameters or combining it with other techniques, such as machine learning, to enhance its performance.

#### 3.2.2. Machine Learning Algorithms

Machine learning has become an essential tool in path planning, with supervised learning and reinforcement learning being two prominent approaches. Supervised learning relies on labeled datasets, where input features, such as obstacle positions or environment maps, are mapped to the corresponding optimal paths or actions. This approach is particularly useful for structured environments or for learning from precomputed solutions, enabling the prediction of efficient and collision-free paths. Reinforcement learning (RL), in contrast, focuses on training an agent to navigate by interacting with the environment and learning through trial and error. By receiving rewards for successful actions such as avoiding obstacles or reaching a goal, the agent discovers the optimal paths in dynamic and uncertain environments. This adaptability makes reinforcement learning especially suited for real-time navigation and complex multi-robot systems.

The application of supervised approaches has been instrumental in advancing robotic path-planning techniques. Recent research has introduced innovative approaches such as the AB-Mapper, which leverages Convolutional Neural Networks (CNN) to enhance path planning [73]. Additionally, trajectory prediction models based on Recurrent Neural Networks (RNN) have been proposed to address complex path-planning challenges, showcasing the adaptability of neural network-based solutions in various robotic contexts [74]. RL algorithms have emerged as a powerful technique in machine learning for addressing path-planning challenges in robotics. Notably, globally guided reinforcement learning approaches have been proposed, demonstrating their potential in optimizing path-planning strategies for various multi-robot systems [77]. State-coded deep Q-network (SC-DQN) algorithms have been effectively applied to unmanned helicopters, highlighting the utility of RL techniques in addressing the unique requirements of specific robotic platforms [78]. Furthermore, RL methods have played a pivotal role in addressing path planning for multiple Unmanned Ground Vehicle (UGV) systems, underscoring their versatility and effectiveness in diverse robotic scenarios [79,80].

The authors of [82] explored reinforcement learning techniques in dynamic obstacle avoidance, offering innovative solutions to challenges in training environments and algorithmic performance. The authors highlighted the limitations of nature-inspired training environments, where skewed probabilities of obstacle encounters led to suboptimal agent learning. To address this, they proposed a traffic-type-independent environment that shifted the focus to high-risk experiences, enabling agents to better handle complex scenarios. This approach, validated through simulations involving mobile robots and maritime ships, demonstrated broad applicability and robustness, even under Gaussian noise and non-linear obstacle behavior. However, the reliance on simulation-based validation raised concerns about the practical implementation in real-world environments, where hardware constraints and unpredictable noise could significantly impact performance. Additionally, while the authors claimed general applicability, a lack of detailed comparisons with alternative training environments limited its ability to benchmark effectiveness, and scalability to multi-robot systems or more complex scenarios remained unexplored.

On the other hand, the authors of [81] shifted the focus to optimizing RL algorithms, particularly the actor-critic framework, to improve dynamic obstacle avoidance in maritime navigation. By enhancing parameter updates and exploration strategies, the study demonstrated improved RL performance in simulated offshore channels under complex meteorological conditions. This optimization addressed challenges that traditional mathematical and swarm intelligence methods faced in defining boundary conditions for real-world scenarios. While the algorithm’s improvements were evident, the narrow focus on maritime applications may limit its generalizability to other domains or dynamic environments. Moreover, the reliance on controlled simulation scenarios did not fully capture the diversity of real-world maritime conditions, such as human-operated vessel interactions, or rapidly changing currents. The absence of comparisons with state-of-the-art RL techniques also left questions about the relative performance of the proposed approach. Including such benchmarks and exploring scalability to broader applications would have strengthened its impact.

#### 3.2.3. Hybrid Techniques

Hybrid techniques have played a significant role in robotic path planning, where they combine optimization algorithms to enhance path planning. For instance, the Global Warm Optimization and Whale Optimization Algorithm (GWO-WOA) hybrid technique [88] has demonstrated its effectiveness in addressing complex path-planning challenges. In another approach, the integration of the Wolf Search Algorithm and the artificial potential field technique (WSA-APF) has shown promising results in path planning and navigation [85]. Similarly, the combination of APF and the Bat Algorithm (BA) (APF-BEA) has been employed to optimize path planning for multi-robot systems [89].

In multi-agent systems, techniques such as the max-min ant colony optimization (ACO) and D* algorithm (max-min ACO-D*) have been adopted to enhance path-planning strategies [90]. The coupling of RRT and APF has been shown to provide effective solutions in dynamic environments [86]. Moreover, the combination of ant colony optimization (ACO) and the Dynamic Window Approach (DWA) has been employed for path planning in multi-robot systems [87]. Additionally, the NPQ algorithm, integrated with RRT*, has demonstrated its utility in path-planning tasks [91].

Other combinations, such as the Artificial Bee Colony (ABC) algorithm and the Electromagnetic Paradigm (EP) (ABC-EP), have been employed for path planning, and their effectiveness is evident in multi-robot systems [92]. Furthermore, the integration of the A* and Dijkstra algorithms has enhanced path planning for robots [93,94]. The genetic algorithm coupled with potential fields has provided autonomous robots with efficient path-planning solutions [95].

While hybrid techniques have proven valuable in enhancing robotic path planning, they also come with their own set of challenges and limitations. One of the main issues with hybrid algorithms, such as GWO-WOA [88] and WSA-APF [85], is the complexity involved in tuning and balancing the parameters of multiple optimization algorithms. These techniques often require fine-tuning of the hybridized components to work effectively, which can be time-consuming and computationally expensive. Moreover, while they provide enhanced performance in certain scenarios, the results may not be consistent across different environments, especially when dealing with dynamic, unpredictable obstacles. Additionally, the integration of algorithms, such as RRT and APF [86] or ACO and DWA [87], introduces complexities in terms of computational cost and real-time application. For instance, while RRT is effective in dynamic environments, it may be slower in highly cluttered spaces, and the addition of APF may not always guarantee optimality, leading to suboptimal solutions. The challenge in such hybrid systems is balancing the benefits of each algorithm without introducing redundant or conflicting behaviors, which could degrade overall system performance.

## 4. Classification Based on Obstacle Consciousness

In this section, a classification framework is introduced, focusing on the consciousness level of dynamic obstacles in path-planning techniques. This classification categorizes obstacles into three distinct groups: conscious obstacles, artificially conscious obstacles, and passive obstacles. By categorizing obstacles based on their level of consciousness, a comprehensive perspective on the challenges posed by different types of obstacles is provided. Conscious obstacles actively respond to the presence of multi-robot systems, demanding path-planning strategies that account for their dynamic and potentially unpredictable behavior. Artificially conscious obstacles are elements deliberately designed or programmed to interact with multi-robot systems, necessitating path-planning approaches that enable communication and coordination. Passive obstacles, on the other hand, require path-planning solutions that prioritize robustness and collision avoidance. This framework enables tailored path-planning approaches to address the unique needs and complexities associated with each category of obstacle in real-world environments. Table 5 reclassifies the techniques based on obstacle consciousness.

### 4.1. Conscious Obstacles

Conscious obstacles can encompass a wide range of dynamic elements that require consideration in path-planning scenarios for different types of multi-robot systems. For UAVs, conscious obstacles might include birds, whose unpredictable flight patterns can pose challenges during flight. On the other hand, pedestrians represent conscious obstacles for UGVs due to their dynamic movements in urban environments. In the case of USVs, a school of fish can be considered a conscious obstacle, as their collective behavior can impact navigation in aquatic settings. Recognizing and accounting for these conscious obstacles is essential for the development of effective and safe path-planning strategies tailored to specific multi-robot systems and their operating environments. For UAVs, relevant research papers include [74,78,97]. Ref. [78] reported on UAVs with a horizontal movement speed of 36 km/h and a vertical movement speed of 1 m/s. In contrast, ref. [74] discussed scenarios involving three quadrotors flying in shared spaces with walking humans, and [97] explored situations with dynamic ground obstacles such as cars, animals, and people during landing. For USVs, ref. [86] is the sole representative. Ref. [86] detailed path planning for ships navigating in the sea.

### 4.2. Artificially Conscious Obstacles

Artificially conscious obstacles encompass elements that have been deliberately designed or programmed to interact with multi-robot systems. Examples include autonomous vehicles in a cooperative network. Recognizing this category underscores the potential benefits of communication and cooperation among elements within an environment. Effective path-planning strategies for these obstacles may involve information exchange and coordination among entities. This can be the easiest scenario to deal with since obstacles are programmable entities and part of the multi-robot system.

Notable works in this category include [40,59,63,66,69,73,85,87,88,98]. The authors of [88] discussed the leader–follower multi-robot system, where robots coordinate their movements to achieve specific goals. In [85], a multi-robot system was proposed for hunting tasks, demonstrating collaborative and adaptive behavior. The authors of [73] presented an approach in which obstacles are governed by the A* algorithm, improving their navigation capabilities. In [66], other robots were treated in a swarm as dynamic obstacles, emphasizing coordination within the group.

Additionally, ref. [63] explored path planning, with dynamic obstacles represented by simple mobile robots. In [87], a prioritized obstacle-avoidance strategy was introduced, ensuring safe navigation for multi-mobile robot systems. The authors of [98] employed a time-varying localization-based priority rule that does not distinguish between agents and obstacles but guarantees safe motion for robots in dynamic environments. Furthermore, in the realm of manipulators, the technique proposed in [35] addresses path planning for a 6-DoF manipulator, focusing on the safe and efficient movement of the robotic arm.

### 4.3. Passive Obstacles

Dynamic passive obstacles are objects in the environment that move without any awareness, intention, or interaction with their surroundings. These obstacles include entities such as falling debris, drifting objects in water or air, and automated mechanisms that lack adaptive intelligence. Their movements are often governed by external forces, such as gravity, wind, or mechanical systems, rather than internal decision-making processes. In path planning, handling dynamic passive obstacles presents unique challenges, as their motion may be unpredictable and influenced by environmental factors, requiring real-time detection and avoidance strategies to ensure safe navigation.

Several significant papers have contributed to this area, such as [32,34,36,39,45,46,49,51,55,56,57,58,61,65,68,70,71,72,76,77,79,89,90,91,92,94,95,99]. The research on UGVs encompasses a range of strategies for challenging path planning. Notable examples include [65], which focused on safety-gap obstacle avoidance, and [76,77], which employed deep reinforcement learning techniques. Ref. [90] explored path planning using the ant colony optimization and D* algorithms, while [95] utilized genetic algorithms and fractional potential fields for navigation. Ref. [49] presented a probabilistic roadmap with self-learning capabilities, and [79] employed evolutionary reinforcement learning. Finally, ref. [45] applied the D* Lite algorithm for robot path planning, and [92] utilized the artificial bee colony algorithm and evolutionary programming. In the context of UAVs, research papers include [47], which delved into autonomous UAV exploration of dynamic environments, and [50], which focused on sense-and-avoid applications. Ref. [52] explored sense-and-avoid systems, enhancing UAV capabilities for safe and effective navigation.

## 5. Classification Based on Communication Style

**Figure 6:**
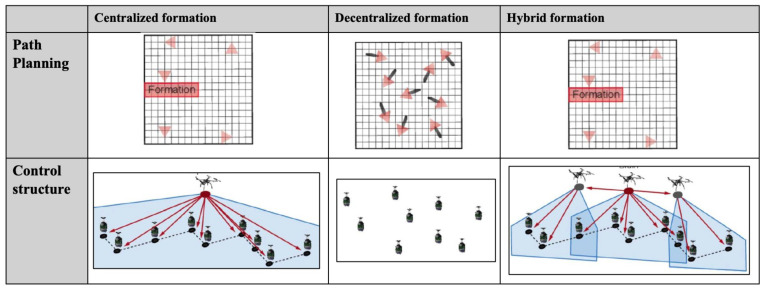
Comparison of centralized, decentralized, and hybrid approaches.

Based on the communication approach, multi-robot systems can be classified into four categories: centralized, decentralized, distributed, and hybrid approaches. Table 6 presents a reclassification of these techniques according to their communication style. Figure 6 shows the comparison of centralized, decentralized, and hybrid approaches. The classification strategy primarily focuses on identifying the entities responsible for processing and controlling the multi-robot system. Each approach has distinct characteristics, as outlined below:

- **Centralized Approach**: In this strategy, a central processing unit, which could be a standalone computer or a robot, assumes full responsibility for controlling and managing the system. This unit processes all data and makes decisions for the entire multi-robot system.
- **Decentralized Approach**: Here, each robot is equipped with its own controller and operates independently based on the data it processes. There is no central entity directing the robots, and each robot’s decisions are made autonomously.
- **Distributed Approach**: In the distributed model, robots collaborate in the system’s planning process. However, unlike the centralized approach, there is no central agent that computes the plans. The responsibility is shared among the robots, with each participating in the decision-making process.
- **Hybrid Approach**: This approach combines multiple communication strategies, integrating elements from the centralized, decentralized, and distributed models. The multi-robot system applies these approaches simultaneously to leverage the advantages of each.

Each approach reflects different organizational structures and levels of autonomy, which are crucial for determining how the robots communicate and work together to achieve system goals. Table 6 further elaborates on these classifications.

**Table 6:** Path-planning papers on dynamic obstacles based on different communication styles.

Type	Robot	Papers	Year	Validation	
				Sim.	Exp.
Centralized	UGV	[32,34,39,56,57,61,65,66,85,88,89,90,91,92,99]	2021, 2022, 2022, 2021, 2022, 2021, 2015, 2022, 2018, 2020, 2018, 2013, 2018, 2018, 2020	[56,57,61,65,66,85,88,89,90,91,92,99]	[32,34,39]
	Heterogeneous	[68]	2021	[68]	
Decentralized	UGV	[40,76,79,98]	2020, 2019, 2017, 2020	[40,76,79,98]	
	UAV	[74]	2021		[74]
Distributed	UGV	[36,59,63,73,77,87]	2019, 2022, 2020, 2020, 2022, 2023	[36,59,63,77,87]	[73]
	UAV	[50,69]	2022, 2021	[50,69]	
	USV	[86]	2022	[86]	
	Manipulator	[35]	2019	[35]	
Hybrid	UGV	[80]	2022	[80]	

### 5.1. Centralized Approaches

Centralized communication can be used for path planning in environments with moving obstacles [32,34,39,56,57,61,65,66,85,88,89,90,91,92,99]. In this scenario, a central control unit collects real-time data from sensors on both the moving objects and the environment, such as cameras or LiDARs, to generate a global map of the environment. The control unit can then use this map to plan a collision-free path for a robot or vehicle while taking into account the movement of the obstacles. The advantage of this approach is that the central control unit has a global view of the environment and can coordinate the movement of multiple objects to avoid collisions and optimize performance criteria, such as travel time or energy consumption [85]. However, a centralized approach also requires continuous communication between the moving objects and the central control unit, which may introduce latency and communication overhead. In recent years, several centralized communication techniques have been proposed for path planning in the presence of moving obstacles, such as model-based optimization, reinforcement learning, and artificial intelligence.

An analysis of centralized communication reveals that it offers a significant advantage in terms of information sharing and collaborative decision making. The central control unit facilitates the seamless integration of data from multiple sources, ensuring consistent and comprehensive information sharing among all entities. This global perspective enhances the ability to make collaborative decisions, such as coordinating robot movements to optimize system-wide objectives and avoid conflicts. However, communication delays or failures could disrupt the flow of critical information, leading to suboptimal decisions or potential collisions. Additionally, the reliance on a single control unit introduces a potential single point of failure, which could impact system reliability and scalability in large, complex environments. These challenges underscore the importance of balancing the benefits of centralized communication with strategies to mitigate its limitations, such as redundancy and robust failure-handling mechanisms.

The centralized communication approach used in the systems proposed in [39,61] effectively manages the path planning and coordination of multiple robots by leveraging global information. In [61], the system utilizes artificial potential fields (APFs) to optimize the trajectories of multiple robots in a shared space, ensuring collision avoidance and coordination. This method benefits from the global perspective, allowing the system to plan paths that are optimal for all agents simultaneously. However, as the number of robots increases, the computational complexity of managing and updating global information grows significantly, as noted in the challenges discussed in [39], where a centralized approach using a modified Dijkstra algorithm for AGVs in a dynamic environment becomes computationally expensive, especially when handling real-time scheduling and collision avoidance. Moreover, both systems face the issue of scalability. In [61], it was shown that as the number of robots increases, the demand on the central controller to continuously compute and adjust paths for all robots may cause delays, impacting the system’s efficiency. Additionally, ref. [39] highlighted the challenge of maintaining real-time performance in large, dynamic environments where rapid re-planning is necessary. Both works also underscore the vulnerability of centralized systems to single points of failure; if the central controller becomes overloaded or fails, the entire system could collapse, leading to a loss of coordination and potential collisions, making these methods less robust in highly complex or large-scale scenarios.

### 5.2. Decentralized Approaches

Decentralized communication is a type of communication in which moving objects in the environment communicate with each other to coordinate their movement without relying on a central control unit. In the context of path planning, decentralized communication can be used to coordinate the movement of multiple robots or vehicles, such as in a swarm robotics system. In this approach, each moving object has its own sensors and local communication capabilities and can share information with its neighboring objects to avoid collisions and optimize its trajectory. The advantage of this approach is that it does not rely on a central control unit, which can reduce latency and communication overhead [76]. However, it also requires each object to have sufficient computational and communication capabilities to perform tasks autonomously. In recent years, several decentralized communication techniques have been proposed for path planning with moving obstacles [40,76,79,98].

Decentralized communication is known for its strengths in scalability, flexibility, and fault tolerance. By distributing decision making across multiple agents, decentralized systems eliminate the single point of failure associated with centralized control, making them more robust in dynamic and unpredictable environments. Information sharing is localized, which reduces the communication overhead and ensures faster response times to environmental changes. Collaborative decision making is achieved through mechanisms such as consensus algorithms or shared objectives, enabling coordinated actions even in the absence of global information. However, challenges include the risk of incomplete or inconsistent information sharing among agents, which can lead to conflicts or suboptimal paths. Furthermore, achieving global optimization through local interactions alone can be difficult, particularly in highly complex environments. These trade-offs suggest that while decentralized communication offers significant advantages, it requires sophisticated algorithms and robust protocols to ensure effective coordination and collision-free navigation.

The authors of [79] employed the Multi-Agent Path Planning with Evolutionary Reinforcement Learning (MAPPER) method, which allows agents to learn navigation policies independently, handling dynamic obstacles and enabling the system to scale effectively with a large number of robots. However, the system can face challenges when agents lack global knowledge, potentially leading to suboptimal decisions. Similarly, ref. [40] utilized decentralized algorithms such as D* Lite, where each robot plans its path based on local data, reducing the computational load and avoiding a single point of failure. Despite these advantages, decentralized methods often struggle with coordination and conflict resolution, especially in environments with many interacting agents or unpredictable obstacles. Both methods highlight the benefits of decentralization, such as scalability and robustness, but also reveal their limitations in complex, dynamic environments, suggesting that hybrid approaches may be necessary to improve coordination and performance.

### 5.3. Distributed Approaches

Distributed communication is a type of communication in which moving objects in the environment communicate with each other through a network of interconnected nodes, without relying on a central control unit. In the context of path planning, distributed communication can be used to coordinate the movement of multiple robots or vehicles, such as in a smart transportation system. In this approach, each moving object has its own sensors and can share information with other objects in its vicinity, which, in turn, can share this information with their neighbors. The advantage of this approach is that it can handle large-scale systems with a high degree of autonomy and flexibility. However, it also requires efficient communication protocols and network infrastructure to ensure reliable and timely communication among the moving objects. In recent years, several distributed communication techniques have been proposed for path planning with dynamic obstacles [36,59,63,73,77,87].

Distributed communication has significant abilities in balancing scalability, robustness, and global coordination. By distributing the workload across multiple agents, the system reduces the risk of a single point of failure while maintaining a level of global coherence in decision making. Information sharing is more extensive than in purely decentralized systems, enabling agents to achieve better coordination and alignment with global objectives. Collaborative decision making is facilitated through structured communication protocols, ensuring that conflicts are resolved and paths are optimized collectively. However, challenges remain, particularly in ensuring timely and reliable communication across the network. Delays or disruptions in communication can lead to inconsistencies in shared data, potentially resulting in suboptimal or conflicting decisions. Additionally, distributed systems require significant computational resources and sophisticated algorithms to manage the complexity of coordination among agents. Despite these challenges, distributed communication offers a promising approach for achieving efficient and resilient path planning in complex, dynamic environments.

In [36], the combination of global path planning using the BAJPSA* algorithm and local path adjustment via the Dynamic Window Approach (DWA) enables real-time adaptation to dynamic obstacles while maintaining the efficiency of the system. However, while this approach offers flexibility and scalability, it faces challenges in collision avoidance, especially in environments with many robots or highly dynamic obstacles. The proposed prioritization method in [36] mitigates these challenges by allowing robots to resolve conflicts based on their assigned priorities, ensuring smoother coordination. Yet, this method can still lead to inefficiencies, particularly when robots with lower priorities are forced to stop or slow down, potentially resulting in delays or suboptimal task execution. Furthermore, the decentralized nature of the system may struggle with ensuring optimality across all robots, as local decision making may not always align with the global objective, leading to potential conflicts or inefficient paths when robots encounter unforeseen environmental changes.

### 5.4. Hybrid Approaches

Hybrid communication is a type of communication that combines both centralized and decentralized approaches, depending on the specific task and environment. In the context of path planning, hybrid communication can be used to coordinate the movement of multiple robots or vehicles in complex environments with moving obstacles. This approach can leverage the advantages of both centralized and decentralized communication, such as global planning capabilities and local responsiveness to changes in the environment. For example, a central control unit can generate a high-level plan for the movement of multiple objects, while each object can use its own sensors and communication capabilities to adapt its trajectory to the local conditions and avoid collisions with moving obstacles. The advantage of this approach is that it can handle complex and dynamic environments with a high degree of autonomy and scalability. However, it also requires efficient communication protocols and coordination mechanisms to ensure reliable and timely communication between the central control unit and the moving objects.

The hybrid communication method used in [80] for multi-robot path planning is centered on deep reinforcement learning (DRL), with a decentralized approach implemented in the execution phase, and a centralized learning mechanism that trains the robots. The system allows robots to operate autonomously based on local observations, mapping laser data directly onto control commands without relying on global maps. This method significantly enhances scalability, as each robot can independently plan its path while interacting with the shared environment using a common policy. However, this decentralized approach faces some challenges in coordination, particularly when robots need to collaborate in complex and dynamic environments. For instance, while robots can navigate efficiently in dynamic environments with obstacles, they may struggle with path conflicts when multiple robots are tasked with reaching their goals simultaneously. The lack of global coordination can lead to inefficient movements, especially in environments with high interaction complexity. Additionally, the absence of real-time communication can result in suboptimal performance, as each robot relies solely on its own sensory data, potentially leading to misalignments in its paths. Despite these challenges, the system’s use of DRL, specifically the Decentralized Proximal Policy Optimization (DPPO) algorithm, offers a promising solution to minimize the total arrival time and improve task efficiency in dynamic environments. This hybrid centralized learning and decentralized execution paradigm offers a balance between autonomy and coordination but requires careful tuning of the reward functions to ensure that robots’ behaviors align with the overall mission goals.

## 6. Classification Based on Obstacle Shape

Path-planning techniques can be categorized based on the shape of the obstacles they are designed to navigate around. There are three primary categories: convex obstacles, non-convex obstacles, and mixed obstacles. Table 7 reclassifies the techniques based on obstacle shape.

Non-convex obstacles represent a class of obstacles that can significantly complicate the path-planning process for autonomous robots. These obstacles exhibit irregular and intricate shapes, making it challenging for traditional path-planning algorithms to find an optimal or collision-free path. In non-convex environments, a critical issue arises when a straight line between two points within the obstacle crosses the obstacle’s boundary. This creates complex, often narrow passages that robots must navigate, increasing the risk of collisions and demanding advanced path-planning strategies. Non-convex obstacles include structures such as buildings, trees, and natural terrain features, which are common in real-world scenarios.

Specialized techniques are required for effective path planning in environments with non-convex obstacles due to the irregular geometry of these obstacles. Traditional methods that assume convexity are often inadequate, failing to generate safe and efficient paths in such complex environments. Path-planning algorithms for non-convex obstacles typically rely on sampling-based planners, such as probabilistic roadmap methods or rapidly exploring random trees, or optimization-based approaches that are more adaptable to irregular shapes. However, optimization techniques, including artificial potential fields (APFs), face challenges in non-convex environments, often becoming trapped in local minima due to the intricate obstacle shapes. A review of the methods presented in [35,39,40,69,74,78,86,90,97] shows that none of these approaches used APF for path planning in the context of non-convex obstacles, as highlighted in the comparison between Table 3 and Table 7.

Non-convex obstacles are prevalent in real-world scenarios, particularly in urban environments, industrial settings, and natural landscapes. Autonomous vehicles, drones, and other robots need to navigate safely in these complex terrains. For autonomous cars, non-convex obstacles include parked vehicles, pedestrians, and complex road junctions. In agriculture, robots must traverse orchards with irregularly shaped trees, and in search-and-rescue missions, intricate debris and collapsed structures are common non-convex obstacles. As a result, the development and implementation of path-planning techniques that effectively address non-convex obstacles are critical for the success of multi-robot systems in a wide range of applications. Addressing the issue of local minima traps in optimization-based methods such as APF is an ongoing challenge in the field of robotics, requiring innovative approaches to overcome this limitation and improve path planning in complex, non-convex environments.

## 7. Empirical Analysis

**Figure 7:**
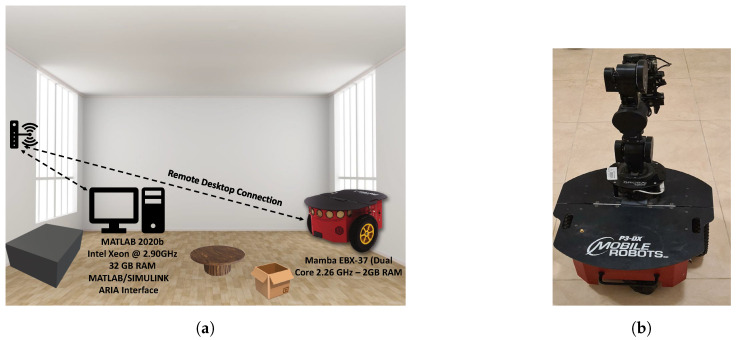
(**a**) Experimental setup. (**b**) The Pioneer P3-DX robot.

This section presents an empirical analysis of different path-planning techniques. In the experiments, a P3-DX mobile robot is controlled through the MATLAB/Simulink 2020b environment via the ARIA interface. The system’s configuration is depicted in Figure 7, which shows the robot connected to a remote desktop running MATLAB 2020b, powered by an Intel Xeon processor at 2.90 GHz with 32 GB of RAM. The robot itself is supported by a Mamba EBX-37 system with a dual-core 2.26 GHz processor and 2 GB of RAM. The experiments involve the robot navigating, with the remote desktop connection allowing for real-time control and monitoring of the robot’s actions. This setup facilitates the evaluation of various path-planning algorithms, providing insights into their computational efficiency, path quality, and real-time performance under controlled conditions.

In addition to evaluating the performance of various path-planning techniques, the experiments were conducted in resource-constrained scenarios, such as with the robot described in the specifications. The robot was equipped with a limited processing unit (Mamba EBX-37 with dual-core 2.26 GHz and 2 GB of RAM) and operated on a battery-powered system. The power constraints further added complexity to the experiments, as the robot was required to perform path planning and real-time navigation while conserving battery life. The robot is capable of running for 8–10 h on three batteries (with no accessories). These limitations necessitate the development of efficient path-planning algorithms that balance performance with the constraints of processing power and energy consumption, reflecting the real-world challenges faced in autonomous robotic systems.

**Figure 8:**
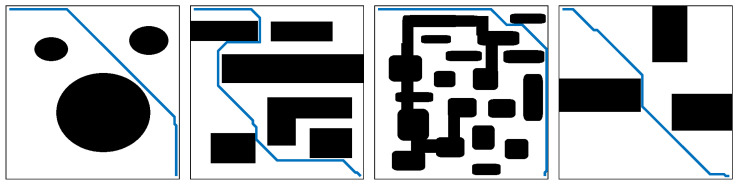
Paths generated using the A* algorithm.

**Figure 9:**
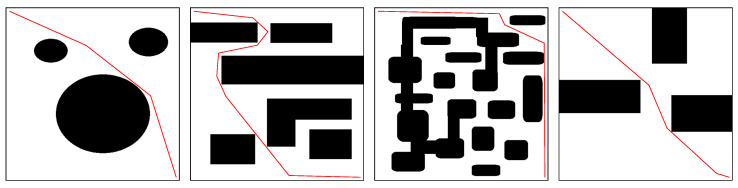
Paths generated using the PRM algorithm.

**Figure 10:**
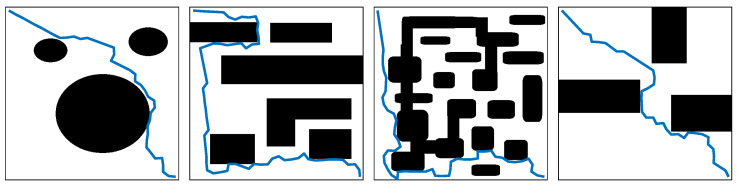
Paths generated using the RRT algorithm.

This section presents the multi-robot path-planning tests conducted on four different maps using the A*, RRT, and PRM techniques. Figure 8, Figure 9 and Figure 10 display the paths obtained using the A*, RRT, and PRM techniques, respectively. Table 8 provides a comparative study of the A* and PRM techniques, focusing on the average path length and processing time. It is well established in the path-planning community that there is a trade-off between the path length and the path quality. The quality of an obstacle-free path is assessed based on two factors: the path length and its smoothness. Achieving a short, smooth path in real time is particularly challenging, especially in resource-constrained environments, where memory, processing power, and time are limited.

Table 8 highlights the time-quality trade-off observed in the results. Shorter paths required longer processing times, as seen in the case of the PRM algorithm, where the shortest path obtained was 102.4 m with a processing time of 4.60 s. In contrast, the A* algorithm paths with a higher grid resolution, indicated by the parameter *G*, generally yielded better-quality paths but also incurred higher computational costs. For example, increasing the grid resolution from 100 × 100 to 250 × 250 increased the processing time from 0.91 s to 2.96 s, while the path length decreased from 108.7 m to 104.6 m. This increase in computational cost highlights the challenges posed by resource constraints in environments where real-time performance is required.

In the case of the RRT algorithm, the step-size parameter *S* showed little impact on the processing time, as indicated in Table 8. However, the RRT algorithm tended to prioritize processing speed over path quality, often resulting in less smooth paths compared to those obtained by the A* and PRM algorithms. This trade-off becomes significant when considering robot controller performance, where smoother paths are easier to follow and require less computational effort for real-time control.

The PRM technique, with its critical parameter being the number of samples *N*, showed that increasing the number of samples resulted in shorter paths but longer execution times. For example, increasing the number of samples from 50 to 200 reduced the path length from 124.7 m to 102.4 m, but the processing time increased from 0.82 s to 4.60 s.

While the RRT algorithm achieved the best processing speed, it produced less smooth paths compared to those obtained by the A* algorithm (Figure 8) and PRM algorithm (Figure 9), which is a significant consideration for controller performance. A smoother path is easier for a robot’s controller to follow, improving overall navigation.

While these results highlight the performance trade-offs in terms of path length and processing time, it is essential to consider the computational complexity of these algorithms in environments with limited resources, such as memory, processing power, and real-time constraints. In particular, the computational burden increases as the resolution of the grid in the A* algorithm or the number of samples in the PRM algorithm increases. These increases in computational load may result in slower processing times, which can be detrimental in real-time systems.

In such environments, optimizing for both execution speed and path quality requires considering more efficient data structures, adaptive techniques, and hybrid approaches that balance these two factors. For example, reducing the grid resolution or using hierarchical methods can help alleviate memory and processing constraints in grid-based methods. Similarly, using adaptive sampling strategies in the PRM method or local planning techniques in the RRT method can reduce the number of samples required while maintaining path quality, thus improving the overall efficiency of path planning in resource-constrained systems.

Additionally, the table compares the performance of two path-planning techniques, artificial potential fields (APFs) and neural fields, using different search window (SW) sizes. The SW parameter refers to the area around the robot within which the potential field is computed at each step toward the goal. The percentage values for the SW represent the dimension of this search window relative to the overall map size.

In resource-constrained environments, where memory, processing power, and time are limited, the choice of the SW size has significant implications. As the SW size increases, both the average path length and processing time typically decrease, which suggests that larger search windows allow for more efficient path planning. However, the computational demand also increases, and this trade-off needs to be carefully managed in systems with limited resources.

For APF, as the SW size increases from 5% to 15%, the path length decreases from 115.47 m to 105.35 m, while the processing time significantly drops from 2.24 s to 0.74 s. This reduction in processing time with larger search windows suggests that APF is more efficient in terms of computation, as it reduces the frequency of recalculating potential fields over smaller areas. However, in a resource-constrained system with limited processing power, using a larger SW size may be less optimal because of the higher computational cost associated with computing potential fields over a larger area, especially in real-time applications.

On the other hand, neural fields show a different pattern, with the smallest SW size (5%) yielding the best balance between the path length (104.83 m) and processing time (0.186 s). This indicates that neural fields perform well with smaller search windows, which may be a crucial advantage in environments with strict memory and processing constraints. As the SW size increases, both the path length and processing time increase, although the computational cost remains lower than that of APF for the same SW sizes.

However, neural network methods require training, which involves the use of large datasets and significant computational resources. The training process can be time-consuming and resource-intensive, especially when dealing with complex environments or high-dimensional data. Additionally, the quality of the trained model depends heavily on the quality and diversity of the training data, making it necessary to collect and preprocess a wide range of samples to ensure that the model generalizes well. Despite these challenges, once trained, neural networks can offer highly efficient and accurate solutions for path planning, as they are capable of learning complex patterns and adapting to various scenarios, without the need for constant recalculation.

## 8. Gaps in Path Planning for Dynamic Obstacles

As illustrated in this review, path planning for dynamic obstacles has seen significant advancements over the years, but there are still several areas where current techniques fall short or lack efficiency. One of the primary gaps lies in real-time path replanning. While many algorithms are designed to update paths dynamically, they often struggle to balance the computational speed needed for real-time operation with the quality of the resulting path. In highly dynamic environments, where obstacles move unpredictably or the landscape changes rapidly, existing approaches frequently fail to recompute optimal paths quickly enough, leading to inefficient or unsafe navigation. This trade-off between computational speed and path quality remains an ongoing challenge.

Another critical gap is the ability to effectively model and incorporate indeterminacy in the motion and behavior of dynamic obstacles. Many current methods rely on probabilistic or stochastic models, but these approaches are often computationally expensive and still fail to fully capture the complexity of obstacle behavior. The inability to integrate robust modeling into the decision-making process limits the reliability of path-planning systems, especially in environments where obstacle behavior is influenced by external factors such as social norms, environmental conditions, or random movements.

Scalability is another significant issue, particularly in multi-robot systems. Coordinating the motion of multiple robots in dynamic environments requires efficient communication, task allocation, and decentralized decision making. As the number of robots increases, the complexity of the interactions and the computational demands grow exponentially. This lack of scalability often leads to suboptimal performance or even system failures in densely populated or highly dynamic settings.

The ability to predict the behavior of dynamic obstacles is another area where path-planning techniques fall short. Many prediction models currently in use are overly simplistic and fail to consider the intricate context of obstacle movement. For instance, pedestrians, vehicles, or animals may move unpredictably due to a variety of factors, and existing models do not adequately account for such nuances. Without accurate predictions, path-planning algorithms cannot reliably avoid collisions or optimize paths.

Handling complex environments also poses a significant challenge. Many current techniques struggle in scenarios with dense, irregularly shaped, or overlapping dynamic obstacles. Environments with narrow passages or limited maneuverability are particularly problematic, as algorithms often fail to find feasible paths or require excessive computational resources to do so. These limitations restrict the applicability of current path-planning methods to more structured and predictable environments.

Energy and resource efficiency are other unresolved issues in the field. While many path-planning techniques prioritize criteria such as collision avoidance or travel time, they often neglect the energy consumption of the planned paths. This oversight is particularly critical for battery-operated robots or vehicles, where efficient energy use is a key operational constraint.

A further gap lies in the absence of standardized evaluation frameworks for path planning in dynamic environments. The lack of consistent benchmarks and metrics makes it difficult to fairly assess and compare different algorithms. Without standardized validation methods, researchers and practitioners cannot reliably determine the effectiveness or efficiency of various approaches, which slows the progression of the field.

Finally, the transition of path-planning techniques from simulation to real-world application remains a significant gap. Many algorithms perform well in controlled environments but struggle when faced with the complexities of real-world deployment. Factors such as sensor noise, unpredictable environmental conditions, and external interferences can significantly degrade the performance of these techniques. Bridging this gap requires robust algorithms that are resilient to real-world challenges and capable of maintaining high performance despite these complexities.

In summary, advancements in dynamic path planning have led to the development of a wide range of techniques that address the complexities of navigating dynamic environments. The current trend in the field emphasizes the integration of artificial intelligence, particularly reinforcement learning, with traditional algorithms to achieve greater adaptability and efficiency in real-time scenarios. Hybrid approaches have gained significant attention, combining the computational efficiency of classical methods such as the A* algorithm with the learning and adaptability capabilities of AI-driven techniques. These trends reflect a shift toward methods that can handle uncertainties, dynamic obstacles, and multi-robot coordination with improved robustness. Despite these advancements, challenges such as scalability, real-time performance, and ensuring global optimization remain active areas of research. The continuous evolution of dynamic path-planning techniques highlights the demand for innovative and reliable solutions in diverse applications.

## 9. Conclusions

This paper presented an in-depth analysis of path-planning techniques for navigating dynamic obstacles in autonomous robotics. By systematically categorizing the existing literature based on path-planning methods, obstacle consciousness, robot communication styles, and obstacle shapes, this study uncovered key insights and emerging trends. It highlighted the need for more resilient and efficient algorithms capable of addressing the challenges posed by diverse and complex obstacle dynamics. Furthermore, the importance of effective communication systems among robots was emphasized as critical for collaborative decision making and optimizing path-planning strategies. The findings stress the necessity of continued research and innovation to address the identified limitations and enhance the effectiveness of dynamic obstacle path planning, ultimately advancing autonomous robot navigation in practical applications.

## Figures and Tables

**Table 1:** Comparison of sensing technologies.

Technology	Range	Resolution	Accuracy	Cost	Application
LiDAR	Long	High	High	High	Autonomous vehicles
Radar	Long	Medium	High	Medium	Collision avoidance
Ultrasonic	Short	Low	Medium	Low	Parking assistance
Infrared	Medium	Medium	Medium	Low	Object detection
Ladar	Long	High	High	High	Mapping
Vision	Medium	High	Medium	Medium	Object recognition

**Table 5:** Path-planning papers on dynamic obstacles based on different types of obstacle consciousness.

Type	Robot	Papers	Year	Validation		Comment
				Sim.	Exp.	
Conscious obstacles	UAV	[74,78,97]	2022, 2021, 2019	[78]	[74,97]	[78] The horizontal movement speed of the flock was 36 km/h, and the vertical movement speed was 1 m/s. [74] Three quadrotors flying in a shared space with walking humans. [97] Dynamic ground obstacles such as cars, animals, and people during landing.
	USV	[86]	2022	[86]		[86] Ships in the sea.
Artificially Conscious Obstacles	UGV	[40,59,63,66,69,73,85,87,88,98]	2022, 2022, 2019, 2022, 2018, 2020, 2022, 2019, 2017, 2021	[40,59,63,66,69,85,87,88,98]	[73]	[88] Leader–follower multi-robot system. [85] Multi-robot system proposed for hunting. [73] Obstacles move based on A* algorithm. [66] Other robots in the swarm are treated as dynamic obstacles. [63] The dynamic obstacles are simple mobile robots. [87] Prioritized obstacle-avoidance strategy for multi-mobile robot systems. [98] Does not need to differentiate between agents and obstacles and uses a time-varying localization-based priority rule to guarantee safe motion.
	Manipulator	[35]	2019	[35]		[35] 6-DoF manipulator.
Passive Obstacles	UGV	[32,34,36,39,45,46,49,51,55,56,57,58,61,65,68,70,71,72,76,77,79,89,90,91,92,94,95,99]	2021, 2021, 2022, 2021, 2020, 2015, 2020, 2021, 2022, 2020, 2018, 2021, 2013, 2014, 2015, 2020, 2018, 2018, 2020, 2020, 2023, 2013, 2018, 2015, 2023, 2017, 2013, 2022	[36,45,46,49,51,55,56,57,58,61,65,68,70,71,72,76,77,79,89,90,91,92,94,95,99]	[32,34,39]	[65] Safety-gap obstacle avoidance. [76] and [77] Deep reinforcement learning. [90] Ant colony optimization and d algorithms. [95] Genetic algorithms and fractional potential fields. [49] Probabilistic roadmap with self-learning. [79] Evolutionary reinforcement learning. [45] D* Lite algorithm for robot path planning. [92] Artificial bee colony and evolutionary programming.
	UAV	[47,48,50,52]	2015, 2022, 2021, 2022	[47,50,52]	[48]	[47] Autonomous UAV exploration of dynamic environments. [52] Sense-and-avoid applications.

**Table 7:** Path-planning papers based on obstacle shape.

Type	Robot	Paper	Year	Validation	
				Sim.	Exp.
Convex	UGV	[32,34,36,56,57,59,61,63,65,66,73,76,77,85,88,89,91,92,98,99]	2021, 2022, 2022, 2021, 2019, 2022, 2022, 2021, 2020, 2015, 2020, 2022, 2018, 2020, 2020, 2018, 2013, 2019, 2020, 2023	[36,56,57,59,61,63,65,66,76,77,85,88,89,91,92,98,99]	[32,34,73]
	UAV	[50]	2022	[50]	
	Heterogeneous	[68]	2021	[68]	
Non-convex	UGV	[39,40,90]	2018, 2018, 2017	[40,90]	[39]
	USV	[86]	2022	[86]	
	UAV	[69,74,78,97]	2022, 2019, 2021, 2021	[69,78,97]	[74]
	Manipulator	[35]	2019	[35]	
Mixed	UGV	[79,80,87]	2022, 2022, 2020	[79,80,87]	

**Table 8:** Comparison of A*, RRT, and PRM techniques based on the average path length and average processing time.

Map	Technique	Parameter	Avg. Path Length (m)	Avg. Processing Time (s)
Map 4	A*	G = 100 × 100	108.7	0.91
		G = 150 × 150	106.3	1.34
		G = 200 × 200	105.4	1.62
		G = 250 × 250	104.6	2.96
	RRT	S = 20	139.5	0.63
		S = 30	134.4	0.60
		S = 40	133.6	0.60
		S = 50	128.5	0.60
	PRM	N = 50	124.7	0.82
		N = 100	123.8	1.90
		N = 150	103.5	3.05
		N = 200	102.4	4.60
	APF	SW = 5%	115.47	2.24
		SW = 10%	111.01	1.08
		SW = 15%	105.35	0.74
	Neural Fields	SW = 15%	114.17	0.58
		SW = 10%	110.25	0.34
		SW = 5%	104.83	0.19

## Data Availability

Data is contained within the article.
